# Effects of Deep Dry Needling on Tremor Severity and Functionality in Stroke: A Case Report

**DOI:** 10.3390/healthcare9010005

**Published:** 2020-12-23

**Authors:** José Antonio Ortín, Elisabeth Bravo-Esteban, Jaime Ibáñez, Pablo Herrero, Julio Gómez-Soriano, Yolanda Marcén-Román

**Affiliations:** 1 Physiotherapy Department, Hospital Universitario Miguel Servet, 50009 Zaragoza, Spain; jaortin@salud.aragon.es; 2 Toledo Physiotherapy Research Group (GIFTO), Facultad de Fisioterapia de Toledo, Universidad de Castilla la Mancha, 45071 Toledo, Spain; Elisabeth.Bravo@uclm.es (E.B.-E.); julio.soriano@uclm.es (J.G.-S.); 3 Department of Bioengineering, Faculty of Engineering, Imperial College London, London SW7 2AZ, UK; jibanezp@ic.ac.uk; 4 Department of Clinical and Movement Neurosciences, Institute of Neurology, University College London, London WC1N 3BG, UK; 5 Department of Physiatry and Nursing, Faculty of Health Sciences, University of Zaragoza, 50009 Zaragoza, Spain; yomarcen@unizar.es

**Keywords:** stroke, dry needling, tremor, functionality, case report

## Abstract

This study aimed to determine the effect of one session of dry needling on the severity of tremor, motor function and skills, and quality of life of a 39-year-old woman with post-stroke tremor. Myofascial trigger points (MTrP) of the following muscles were treated: extensor digitorum, flexor digitorum superficialis and profundus, brachioradialis, short head of biceps brachii, long head of triceps brachii, mid deltoid, infraspinatus, teres minor, upper trapezius, and supraspinatus. Outcomes were assessed via (i) clinical scales (activity of daily living (ADL-T24), a visual analog scale (VAS), and the Archimedes spiral), (ii) a functional test (9-Hole Peg test), and (iii) biomechanical and neurophysiological measurements (inertial sensors, electromyography (EMG), and dynamometry). The subject showed a decrease in the severity of tremor during postural (72.7%) and functional (54%) tasks after treatment. EMG activity decreased after the session and returned to basal levels 4 days after. There was an improvement post-intervention (27.84 s) and 4 days after (32.43 s) in functionality and manual dexterity of the affected limb, measured with the 9-Hole Peg test, as well as in the patient’s hand and lateral pinch strength after the treatment (26.9% and 5%, respectively), that was maintained 4 days later (15.4% and 16.7%, respectively).

## 1. Introduction

Cerebrovascular accidents (CVA) comprise one of the main causes of impairment worldwide [1]. Around 50% of CVA survivors suffer from severe impairments that directly affect their quality of life and functionality [1,2], of which approximately 50% cannot use the affected hand in daily life activities [3]. Hypertonia is one of the most frequent complications in stroke patients (4–42.6%) [4], while tremor is one of the most disabling symptoms [5].

Myofascial trigger points (MTrPs) are hypersensitive areas of the skeletal muscle, associated with palpable nodules in the taut bands of muscle fibers. They have a high prevalence in patients following a stroke and are moderately associated with pain and function [6,7]. Dry needling (DN) is one of the most employed and effective methods for treating MTrPs [8] and has shown to be effective to decrease spasticity in sub-acute [9] and chronic stroke patients [10,11]. Although the mechanism of action of DN is still unclear, it is known that DN inhibits the H-reflex [12], decrease the frequency of motor unit spontaneous firing spikes [13] and may modify the contractile capacities of the spastic muscle [14]. Recent studies have also shown that DN achieves an increased activation of the sensory and motor areas in post-stroke [15,16]. Moreover, some experimental studies in animal models have shown that DN reduces the abnormal electromyographic (EMG) activity that is characteristic of MTrPs [17] and that an intact afferent pathway and normal spinal cord function are needed to evoke remote effects of dry needling on EMG endplate noise [18].

To the best of our knowledge, there are no studies analyzing the effects of DN on tremor. However, according to the aforementioned studies, DN could be useful for normalizing movement disorders, such as tremors, which are directly related to EMG alterations. Therefore, the aim of this study is to research the effect of one session of DN on the severity of tremor of a patient with chronic stroke.

## 2. Materials and Methods

### 2.1. Patient Information

A 39-year-old woman that suffered a right temporo-parietal ischemic stroke 14-years ago took part in this study. The stroke episode resulted in hemiplegia to the left upper limb that was characterized by a slight tremor when the arm was at rest, and became more severe and frequent when performing intentional activities, especially during open kinetic chain exercises. Eventually, the patient regained her previous functionality, mobility, and the ability to perform most daily life activities compared to her former condition, but the level of tremor severity remained constant. She has been regularly treated with good overall results, but with poor improvement in tremor severity. The cognitive capacity of the patient is good, and she is currently working as a nurse. The patient was informed of the aim and characteristics of the study and voluntarily signed the consent form to participate.

### 2.2. Design and Assessment

A pretest–posttest study was designed [19], with assessments at three different time points: pre-intervention to record baseline values (pre), immediately after the intervention (post), and 4 days after (post-4d). All measurements were taken under the same conditions and in the same room. A protocol was established for evaluating the tremor severity, motor function and quality of life. This included the following: (i) clinical scales (activity of daily living scale (ADL-T24), a visual analog scale (VAS), and the Archimedes spiral), (ii) a functional test (9-Hole Peg test), and (iii) biomechanical and neurophysiological measurements of the upper limb tremor (inertial sensors, electromyography (EMG), and dynamometry).

### 2.3. Clinical and Functional Assessment

The patient’s clinical assessment was performed using the ADL-T24 [20] scale, which records difficulties in performing 8 daily life activities. Item 4 “shaving” was removed and item 6 “reading a book” was modified to specify that forearms had to be supported. The Archimedes spiral is considered an integral component of tremor evaluation routines [21] and has been used in clinical trials to quantify essential tremor [22,23]. The spiral was drawn on the Temblores^®^ tablet application (Temblores APP, Zaragoza, Spain), using the index finger of the affected hand on a template after placing the finger in the spiral’s center with the assessor’s help.

Functional assessment was performed using the 9-Hole Peg test (9HPT), which quantifies the time (in seconds) it takes to place and remove 9 pegs on a board. This test has high inter-examiner and moderate intra-examiner reliability [24]. Both ADL-T24 and 9HPT are effective tools for the evaluation of tremor severity [25].

A subjective assessment of the treatment effect on the patient’s tremors was also measured with a Likert-type scale on satisfaction, with 7 possible responses (1 = “very unsatisfied”, 2 = “unsatisfied”, 3 = “slightly unsatisfied, 4 = “neutral”, 5 = “slightly satisfied”, 6 = “satisfied”, and 7 = “very satisfied”), and on a VAS (score from 0 to 10 where the patient assesses her perception) [26].

### 2.4. Biomechanical and Neurophysiologic Assessment

Dynamometry was employed on the affected wrist using an homologated hydraulic dynamometer (Baseline 12-0241 LITE Hydraulic Hand Dynamometer^®^, Warwick, RI, USA), with the patient sitting, elbow flexed at 90°, forearm in semi-pronation, wrist extended at 0–30°, and 15° of ulnar deviation [27]. A pinch gauge dynamometer (Baseline^®^ Hydraulic Pinch Gauges, Warwick, RI, USA) was used for the dynamometry of the thumb lateral pinch. Three measurements were recorded in both cases, and the arithmetical mean was obtained [28,29].

**Figure 1:**
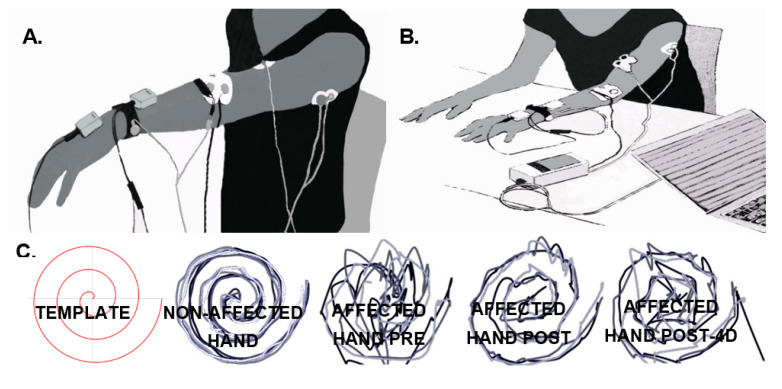
Subject’s posture and position of the sensors for tremor recording using gyroscopes (grey boxes) and electromyography (EMG) (white circular electrode) during the postural task (**A**) and before starting the functional task (9-Hole Peg test (9HPT)). (**B**,**C**) Superimposition of the Archimedes spiral drawn on a template after placing the finger in the spiral’s center with the assessor’s help with the index finger of the non-affected hand, and the affected hand before (pre), after the intervention (post), and 4 days after the intervention (post-4d), from left to right.

Tremor of the left arm was measured with solid-state gyroscopes and surface EMG, while the patient maintained the arms outstretched against gravity for 60 s (Figure 1A) (shoulders flexed at 90°, and elbows and wrists extended) and also while performing a functional task (the execution of the 9HPT, as described in Figure 1B). Two gyroscopes (Technaid S.L., Madrid, Spain) were placed on the dorsum of the hand and on the distal third of the forearm to measure wrist flexion–extension tremors by computing the difference between them. The data resulting from the subtraction were high-pass filtered (Butterworth, fc = 0.1 Hz, 3rd order) and sampled at 50 Hz [30]. The level of the wrist tremors was calculated by computing the power spectral density (PSD) of the resulting signal (Welch’s method, sliding windows of 2 s, no overlapping, 0.5 Hz resolution). The severity of the tremors was defined as the PSD peak value around the tremor’s frequency (3–4.5 Hz). The results of the previous calculations were used to compare the tremors in the different experimental phases [30].

Surface EMG was recorded using bipolar electrodes placed on the muscle belly of (i) wrist extensors, (ii) wrist flexors, (iii) triceps, and (iv) biceps brachii. A humidified bracelet was attached to the wrist and used as common reference. The data were amplified and sampled at 250 Hz (Trentadue, OT Bioelettronica, Torino, Italy). EMG signals were first band-pass filtered (Butterworth, 0.5 < f < 30 Hz, 3rd order), and then the PSD was estimated (Welch’s method, sliding windows of 2 s, no overlapping, 0.5 Hz resolution). Tremor severity was defined as the area under the PSD function around the tremor frequency (3–4.5 Hz) relative to the total area under the PSD [31].

### 2.5. Intervention

DN was applied in a single session by a specialized physiotherapist that employed solid, non-beveled, filiform DN needles of 0.25 × 25 and 0.32 × 40 mm. Muscles whose co-contraction was considered as a possible cause of the tremor and those with visible muscle activity at rest were treated unilaterally and in a distal-to-proximal-chronological order. The muscles that met these criteria were (i) extensor digitorum, (ii–iii) flexor digitorum superficialis and profundus, (iv) brachioradialis, (v) short head of biceps brachii, (vi) long head of triceps brachii, (vii) mid deltoid, (viii) infraspinatus, (ix) teres minor, (x) upper trapezius, and (xi) supraspinatus. The treatment was performed following the technique specified by the Dry Needling for Hypertonia and Spasticity (DNHS) [6,32], with needling intensity adjusted to the patient’s pain tolerance through oral feedback. Furthermore, the patient was instructed to say “stop” if she needed a break at any point. Local twitch responses were obtained from every treated muscle to confirm that the MTrP was needled [33].

## 3. Results

The subject showed a decrease in the severity of tremor during postural (72.7%) and functional (54%) tasks after treatment, which was maintained only in the case of postural tasks 4 days later (73.6%). In the case of EMG, the overall activity decreased after the session and returned to basal levels 4 days later. In agreement with these results, there was an improvement post-intervention (27.84 s) and 4 days after (32.43 s) in functionality and manual dexterity of the affected limb, measured with the 9-Hole Peg test (Table 1). There was also an improvement in the patient’s hand and lateral pinch strength after the treatment (26.9% and 5%, respectively), which was maintained 4 days later (15.4% and 16.7%, respectively).

DN was well tolerated, and no adverse effects were observed. The patient reported a generalized feeling of heaviness in the upper limb immediately after the intervention, which improved after several hours. Table 1 shows the data from the clinical and functional assessment. It also includes the results obtained from gyroscopic and EMG recordings for tremor evaluation, both at rest and while performing functional tasks.

Subjective perception of the treatment effect on tremors, measured on a Likert-type scale, obtained a score of 4 (“neutral”) post-intervention, and of 5 (“slightly satisfied”) at post-4d. Figure 1C shows, in qualitative terms, the outcome on kinetic tremor measured by drawing the Archimedes spiral. An improvement is observed immediately after the intervention that slightly decreases at post-4d.

## 4. Discussion

This is the first study to research and quantify the effect of DN on the severity of tremor in the upper limb of a patient after a stroke. Results showed a decrease in the tremor severity after a single session of DN that persisted for 4 days. Tremor reduction was registered with gyroscopic and muscle activity recordings and with clinical scales measuring the functionality and dexterity of the affected upper limb. No significant clinical changes were observed in the patient’s perception or in the self-reported impact of the tremors on the ability to carry out daily life activities.

Tremor reduction, as evidenced by the Archimedes spiral test and the recordings using gyroscopes, is similar to that reported by other studies using botulinum toxin A (BTX-A) injection [34,35]. Although there are no studies that compare DN with BTX-A injection techniques, both could rely on similar mechanisms of action, namely, destroying dysfunctional motor plates and preventing the release of acetylcholine as a result of mechanical breaking in the case of needling [36] or chemical denervation in the case of BTX-A injections [37]. However, studies on BTX-A injections have not revealed significant changes in functionality [38,39], whereas the patient in this study experienced a decrease of 25% in the time spent to complete the 9HPT, which persisted for 4 days after the intervention, despite the fact that both BTX-A and DN seem to have central effects [15,16,40].

Unlike the lateral pinch, the patient’s grip strength increased. This could be due to a greater effect on the proximal muscles and/or a decrease in the EMG activity of the antagonist muscles. Importantly, previous studies reported that BTX-A injections led to a decrease in EMG activity, which was associated with strength loss in both the agonist and antagonist muscles—this is even considered an adverse secondary effect [38,39].

It is difficult to determine the clinical significance of the results despite finding quantifiable improvements in the patient’s tremor. Nevertheless, muscles involved in the wrist tremor (where a higher impairment was evident at the beginning of the study) showed improvements in functionality (at least 25%), gyroscopic recordings at rest and while performing intentional tasks, grip strength, and EMG. On the other hand, the changes observed post-treatment for grip strength, accelerometry of intentional tremor, and EMG variables disappeared or dropped at post-4d. A decrease in the effectiveness of DN on the patient’s tremor 4 days after the intervention was also found in the Archimedes spiral test. These data suggest that repeated treatment sessions could increase and prolong the effectiveness of the proposed technique. This study has also limitations; for example, it is only one case and, thus, does not allow for the establishment of a cause–effect relationship between the use of DN and the improvement of tremor. Moreover, the patient only received a single session of DN, and its effects were only measured in the short term (4 days after DN). Further studies including a control group with more DN sessions and a longer follow-up must be performed in order to evaluate if DN is effective to improve tremor and the duration of its effects.

## 5. Conclusions

A single session of DN was effective to improve tremors in a patient with chronic stroke, although a cause–effect relationship cannot be established due to the study methodology. Although the patient did not report significant subjective changes, the treatment achieved an objective decrease in tremors that persisted for at least 4 days. These results must be interpreted with caution, and further research with larger sample sizes, more DN sessions and a control group needs to be conducted.

## Figures and Tables

**Table 1:** Clinical, functional, biomechanical and neurophysiological outcomes. Percentage of change post-intervention (post) and 4 days later (post-4d) compared to the basal state (pre).

Outcomes/Assessments	Pre	Post	Post-4d	% of Change Post	% of Change Post-4d
Clinical and functional outcomes					
9-Hole Peg test (s)	111.13	83.29	78.70	−25.1	−29.2
VAS (0–10)	4	3	6	−10.0	+20.0
Hand-grip dynamometry (Kg)	26.0	33.0	30.0	+26.9	+15.4
Pinch dynamometry (Kg)	6.0	6.3	7.0	+5.0	+16.7
ADL-T24 (0–21)	8	8	8	0	0
Tremor during postural task					
Gyroscopes (rad/s)^2^/Hz	0.220	0.060	0.058	−72.7	−73.6
EMG wrist extensors (relative tremor power)	0.414	0.071	0.388	−82.9	−6.3
EMG wrist flexors (relative tremor power)	0.412	0.397	0.275	−03.7	−33.3
EMG triceps (relative tremor power)	0.061	0.034	0.004	−44.3	−93.5
EMG biceps (relative tremor power)	0.019	0.061	0.029	+321.0	+52.6
Tremor during functional task					
Gyroscopes (rad/s)^2^/Hz	0.689	0.317	0.630	−54.0	−08.6
EMG wrist extensors (relative tremor power)	0.094	0.075	0.079	−20.2	−16.0
EMG wrist flexors (relative tremor power)	0.061	0.029	0.108	−52.4	+77.0
EMG triceps (relative tremor power)	0.064	0.028	0.061	−56.3	−04.7
EMG biceps (relative tremor power)	0.021	0.015	0.034	−28.6	+61.9

VAS: visual analogue scale; ADL: activity of daily living.
